# Religious Fundamentalism, Satisfaction with Life and Posttraumatic Stress Symptoms Intensity in a Polish Sample of People Living with HIV/AIDS

**DOI:** 10.1007/s10943-018-0615-1

**Published:** 2018-04-07

**Authors:** Włodzimierz Oniszczenko, Marcin Rzeszutek, Ewa Firląg-Burkacka

**Affiliations:** 1 Faculty of Psychology, University of Warsaw, Stawki 5/7, 00-183 Warsaw, Poland; 2 Warsaw’s Hospital for Infectious Diseases, Warsaw, Poland

**Keywords:** Trauma, Religious fundamentalism, Satisfaction with life, HIV/AIDS

## Abstract

We investigated the relationship between religious fundamentalism, satisfaction with life and the intensity of posttraumatic stress symptoms in people living with HIV/AIDS. The study was conducted on 283 adults, including 242 HIV-positive patients and 41 individuals with AIDS, aged from 20 to 74. Religious fundamentalism was positively correlated with age and posttraumatic stress symptoms intensity. Negative correlation between satisfaction with life and posttraumatic stress intensity was also found. Religious fundamentalism and satisfaction with life accounted for 34% of the variance in posttraumatic stress symptoms intensity. The level of patients’ education mediated the relationship between religious fundamentalism and the posttraumatic stress symptoms intensity.

## Introduction

HIV infection is a serious social and medical problem. Thanks to the effectiveness of antiretroviral therapy, HIV infection has become a chronic disease (Deeks et al. 2013). People living with HIV (PLWH) as well as people living with HIV/AIDS (PLWHA) may experience multiple forms of stigma, all of which can negatively impact their health, quality of life, social support and well-being (Logie and Gadalla 2009; Slater et al. 2015). In addition, HIV-positive individuals may suffer from many psychiatric disorders such as anxiety and depression (Kee et al. 2015; Robertson et al. 2014), sleep disturbance (Leyro et al. 2014), substance abuse (Chander et al. 2006) and HIV-related fatigue (Barroso et al. 2010).

PLWHA and PLWH report high rates of exposure to past or current traumatic events and high levels of posttraumatic stress symptoms (PTSSs). Several other studies suggest three major sources of posttraumatic stress in HIV-positive individuals: psychosocial trauma prior to HIV infection (e.g., childhood abuse, rape, physical assault, partner-inflicted violence) (LeGrand et al. 2015), receiving a diagnosis of the presence of HIV and the related perceived threat to life and cumbersome treatment (Martin and Kagee 2011) and the stigmatization of HIV-positive individuals (Bogart et al. 2011; Breet et al. 2014; Ye et al. 2015).

The presence of health and mental health problems and PTSSs may influence psychological well-being, including satisfaction with life, in both PLWH and PLWHA. Oberjé et al. (2015) showed that environmental well-being (i.e., satisfaction with the conditions and safety of one’s living place) and mental health serve as main predictors of subjective well-being (SWB) in HIV-positive individuals, while physical health, social well-being and financial well-being explain SWB to a lesser extent. Satisfaction with life is the cognitive aspect of SWB that refers to a conscious cognitive judgment of one’s life. Satisfaction with life reflects the degree to which an individual judges the overall quality of his or her life as a whole in a favorable way (Diener et al. 1985). It is noteworthy that a wide range of factors such as physiological, psychological, social, spiritual and economic elements may determine well-being (Noehammer 2013). In an earlier study, Eller and Mahat (2007) showed that anxiety level, health transition, physical role, physical function and mental health may explain life satisfaction in HIV-positive women (see also Smith et al. 1998). Greeff et al. (2010) showed that perceived HIV-related stigma has significant negative impact on life satisfaction. Rzeszutek et al. (2015) showed that longer HIV infection duration may intensify PTSSs, but perceived availability of social support may be a buffer against HIV-related trauma symptoms in PLWHA. Other studies have shown that treatment adherence (Drozd et al. 2014), a web-based intervention trial in patients with chronic HIV infection (Reis et al. 2013) and optimism (Ammirati et al. 2015) may have a positive impact on SWB in PLWH.

Some authors suggest that spiritual and religious factors may contribute to high levels of satisfaction with life in PLWHA (Cotton et al. 2006). Liboro and Walsh (2015) examined the lived experiences and personal perspectives of men living with HIV/AIDS and suggested that religions such as Catholicism can promote acceptance and support for greater well-being of men living with HIV/AIDS. In particular, religiosity may manifest itself in religious beliefs or attitudes and participation in religious services or personal acts such as prayer. Positive association between religiosity and well-being in PLWHA is based on religion providing a source of social support, recovery of meaning in life and a coping mechanism (Siegel and Schrimshaw 2002; Steglitz et al. 2012). Positive religious coping (e.g., a reflection of a secure relationship with God, a belief in life’s larger meaning and a sense of spiritual connectedness with others; Pargament et al. 1998) was significantly associated with positive mood and life satisfaction in PLWH (Lee et al. 2014), reduced depressive symptoms in PLWHA (Dalmida et al. 2013) and PTSSs in HIV-positive women (Brownley et al. 2015). Religiosity may also be associated with lower-HIV-risk behaviors (Shaw and El-Bassel 2014) and adherence to therapy in PLWH (Kisenyi et al. 2013).

Religious fundamentalism is not typical of any particular religion and has its manifestations across diverse religions. Most research on religious fundamentalism indicates its negative implications for the PLWH (e.g., people living with HIV and AIDS are often viewed as “sinners” and of low moral standards). Nevertheless, it should be noted that the activities of fundamentalist churches and religion organizations was an important factor in the effort to control the epidemic and to care for those affected by it (Jonsen and Stryker 1993). In her work, Szaflarski (2013) showed the power of faith communities (esp. among African-Americans) to influence HIV-risk behaviors and attitudes toward PLWH, as well as a role of faith-based community interventions to reduce stigma and enhance HIV prevention/care.

According to Altemeyer and Hunsberger (1992, p. 118), religious fundamentalism is a cognitive process corresponding to “the belief that there is one set of religious teachings that clearly contains the fundamental, basic, intrinsic, inerrant truth about humanity and deity; that this essential truth is fundamentally opposed by forces of evil which must be vigorously fought; that this truth must be followed today according to the fundamental, unchangeable practices of the past; and that those who believe and follow these fundamental teachings have a special relationship with the deity.”

To date, no empirical studies have been conducted to test the relationship between religious fundamentalism and PTSS in PLWHA. Religious fundamentalism focuses on the certainty that one’s religious beliefs are correct and the belief that one has access to absolute truth. Ness and Wintrob (1980) showed that people who participated in fundamentalist religious activities were less likely to report emotional distress. Sethi and Seligman (1993) demonstrated that fundamentalist individuals were significantly more optimistic when compared to religiously moderate and liberal groups. The greater optimism of fundamentalist individuals may be entirely accounted for by the greater hope and daily influence fundamentalism engenders, along with the greater optimism of the religious services they hear. Green and Elliott (2010) suppose that the strict worldview of religious fundamentalists reduces their uncertainty and provides them with a stable, optimistic framework that helps them to understand and cope with life’s difficulties. According to Hood et al. (2005), religious fundamentalism is a way of coping, understood as a search for meaning that helps fundamentalists meet several personal needs for meaning, such as purpose, value, efficacy and self-worth in stressful situations.

Although AbdAleati et al. (2016) suggest that religiousness may serve as a protective factor for physical and mental health, religious fundamentalism is not recognized as a factor directly associated with human health. However, Asp et al. (2012) found that patients with bilateral damage to the ventromedial prefrontal cortex reported significantly high level of religious fundamentalism. In another study, Phillips and Ano (2015) showed that religious fundamentalism is associated with a number of coping strategies that facilitate adjustment to stressful situations, such as illness. Błażek and Besta (2012) demonstrated that religious fundamentalism is highly correlated with intrinsic religiosity. It has been shown (Hui and Coleman 2013; Sanders et al. 2015) that intrinsic religiousness is inversely correlated with depression and anxiety, including death anxiety in older people. Moreover, it was shown that intrinsic religiosity is a modest protective factor against death anxiety among PLWHA (Miller et al. 2012) and decreases PTSS in people exposed to terror (Laufer and Solomon 2011). In turn, Aydin et al. (2010) showed that socially excluded individuals report significantly higher levels of religious affiliation and stronger intentions to engage in religious behaviors compared to non-excluded individuals. This result suggests that stigmatized and traumatized HIV-positive persons may also exacerbate the attitude of religious fundamentalism when trauma arises.

Several authors found that people suffering from lifetime trauma (Krause 2004; Triplett et al. 2012), having PTSD symptoms (Besser and Neria 2009; Karatzias et al. 2013), suffering from a traumatically acquired disability (Hernandez et al. 2014) or perceiving severe stress (Kaya et al. 2015) generally experience less satisfaction with life. Only a few studies refer to the relationship between religious fundamentalism and life satisfaction. Some data suggest that religious fundamentalism has a weak positive correlation with life satisfaction in Catholic undergraduate students (Carlucci et al. 2015) and South African social science students, their family members and friends (Nell 2014).

The aim of this study was to investigate the relationships between religious fundamentalism, satisfaction with life and PTSS in PLWHA. Taking into account the aforementioned findings, we hypothesized that: (a) religious fundamentalism would be negatively correlated with the PTSS; (b) satisfaction with life would be negatively correlated with PTSS; and (c) religious fundamentalism would be positively correlated with satisfaction with life.

## Method

### Participants

The sample consisted of 283 adults (250 men and 33 women) with a clinical diagnosis of HIV infection. Participants’ ages ranged from 20 to 74 (*M* = 38.72; SD = 9.39). There were 242 patients with a clinical diagnosis of HIV positive and 41 individuals with AIDS. All individuals were treated at the Warsaw’s Hospital for Infectious Disease. The duration of the HIV infection in the entire sample ranged from 1 to 33 years (*M* = 7.90; SD = 7.05). In the studied group, 123 participants had higher education, 99 participants had secondary education, and 61 participants had primary education. Participants were recruited directly by the psychologists who conducted the study. All self-report questionnaires were administered in a standard manner.

The study was anonymous, and participation was voluntary. Informed consent was obtained from all patients before they were included in the study, and participants were not remunerated.

### Measures

The quantitative level of trauma symptoms was assessed with the PTSD Factorial Version Inventory (PTSDF) (Strelau et al. 2002). The PTSDF has 30 items. Patients were asked to report how often in the past several months they experienced a given thought, behavior or emotion related to the traumatic event. Participants gave answers by selecting one of four options: never, rarely, often and always. The total score was calculated as a sum of answers to all 30 items, scoring from 0 to 3 points. This questionnaire has two subscales corresponding to two basic PTSD factors (American Psychiatric Association 1994): intrusion/arousal (Cronbach’s alpha = 0.95) and avoidance/numbing (Cronbach’s alpha = 0.93). Both subscales are highly correlated with each other (*r* = 0.81). Scores on each subscale were added to calculate the general intensity of trauma symptoms (general scale: Cronbach’s alpha = 0.96). All Cronbach’s alphas given in parentheses are derived from the present sample of HIV-positive individuals. All analyses in the reported studies were based on the total score of the PTSDF inventory. Higher scores in the PTSDF general scale indicate higher levels of PTSSs.

Religious fundamentalism was assessed with the 20-item religious fundamentalism scale (RFS) (Altemeyer and Hunsberger 1992; Polish adaptation, Tomasz Besta and Magdalena Błażek). The scale measures religious fundamentalism understood in terms of a structure of religious attitudes. Each item is rated on an anchored scale from − 4 (*very strong disagreement*) to + 4 (*very strong agreement*). The higher the score, the higher the religious fundamentalism. Cronbach’s alpha coefficient for the Polish version of the RFS in the present sample was 0.89.

The participants’ satisfaction with life was measured using the Satisfaction with Life Scale (SWLS) (Diener et al. 1985; Polish adaptation, Zygfryd Juczyński). The SWLS has five items. Respondents indicate the degree of agreement with each item on a 7-point scale ranging from 1 (*strongly disagree*) to 7 (*strongly agree*). Thus, a higher total score on this scale indicates greater satisfaction. Cronbach’s alpha coefficient for the Polish version of the SWLS in the present sample was 0.89.

### Statistical Analysis

The statistical analysis was performed with IBM SPSS Statistics 22 (IBM Corp. 2013). Descriptive statistics such as the means and standard deviations of the main variables are reported. Since the compared groups were not equinumerous, the Mann–Whitney *U* test was used to test the significance of differences between HIV-positive and HIV/AIDS patients. Relationships among variables were examined with the Pearson product-moment coefficients.

Validity of religious fundamentalism and satisfaction with life as predictors of trauma intensity was estimated by means of multiple regression analysis.

## Results

Table 1 shows the means and standard deviations for age, trauma-related symptoms intensity, religious fundamentalism and satisfaction with life separately for HIV-positive and HIV/AIDS patients. The two groups were compared using the Mann–Whitney *U* test. Compared with HIV-positive individuals, HIV/AIDS patients had significantly lower levels of satisfaction with life.

**Table 1:** Mean and standard deviation comparisons for age, trauma symptom intensity, religious fundamentalism and satisfaction with life in PLWH (*n* = 242) and PLWHA (*n* = 41)

Variables	PLWH *M* (SD)	PLWHA *M* (SD)	*Z*	Cohen’s *d*
Age	38.29 (9.32)	41.29 (9.55)	− 1.95	− 0.32
Trauma symptom intensity	58.76 (19.49)	60.28 (17.74)	− 0.66	− 0.08
Religious fundamentalism	78.93 (28.51)	72.60 (28.72)	− 1.09	0.22
Satisfaction with life	18.72 (6.93)	15.24 (7.72)	− 2.95**	0.47

*Z* = value for the Mann–Whitney *U* test

**p < 0.01

Table 2 presents the correlations among age, trauma symptom intensity, religious fundamentalism and satisfaction with life in the whole sample. Positive correlations were found for religious fundamentalism and age as well as religious fundamentalism and trauma symptoms intensity. Negative correlation between satisfaction with life and trauma symptom intensity was also found.

**Table 2:** Pearson’s *r* correlations among age, HIV infection duration, trauma symptom intensity, religious fundamentalism and satisfaction with life in the whole sample (*n* = 283)

	Trauma intensity	Religious fundamentalism	Satisfaction with life
Age	0.04	0.12*	− 0.01
Trauma symptom intensity		0.25**	− 0.56**
Religious fundamentalism			− 0.07

*p < 0.05; **p < 0.01

To determine the extent to which religious fundamentalism and satisfaction with life can be viewed as predictors of the trauma symptoms intensity (treated as the explained variable in the analysis) among participants, we conducted a multiple regression analysis. The results are shown in Table 3.

**Table 3:** Multiple regression analysis of religious fundamentalism and satisfaction with life as predictors of trauma symptom intensity in PLWHA (*n* = 283)

Variable	*B*	SE *B*	*ß*	Semi-partial correlations
Religious fundamentalism	0.14	0.03	0.21***	0.20
Satisfaction with life	− 1.48	0.13	− 0.55***	− 0.55

*SE* standard error

***p < 0.001

As shown in Table 3, the religious fundamentalism explained 4% and satisfaction with life explained 30% of the global trauma symptoms. Together these two variables accounted for 34% of the variance of the global trauma symptoms.

**Fig. 1:**
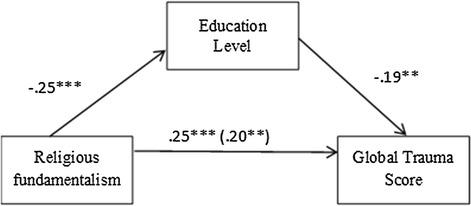
Relationship between religious fundamentalism and the global trauma score as mediated by education level among participants. *Note* ***p < .001; **p < .01

The additional ad hoc assumption was that the education level would mediate the relationship between the religious fundamentalism and the global trauma score among participants (see, for example, Schwadel 2016). Three separate regression analyses were conducted following the Baron and Kenny approach (1986). To assess for mediation, the Sobel test for mediation was performed. The results showed that education level was a significant, partial mediator of the relationship between the religious fundamentalism and the global trauma score (Sobel test values: *Z* = 2.563, *p* = 0.0010). These results are presented in Fig. 1.

The mediation analysis (see Fig. 1) revealed that the total effect between the religious fundamentalism and the global trauma score (*ß* = 0.25, *p* < 0.001) was reduced upon the inclusion of the mediator—participants’ educational level (*ß* = 0.20, *p* < 0.01).

## Discussion

The aim of this study was to investigate the relationships among religious fundamentalism, satisfaction with life PTSSs and PTSSs in PLWHA.

Religious fundamentalism was weakly positively associated with PTSSs (see Table 2). It seems that increase in PTSSs level is conducive to intensification of fundamentalist religious attitudes, which are related to religious behavior. The result is surprising, as we assumed that religious fundamentalism will be negatively correlated with PTSSs, as many other studies suggested (Hood et al. 2005; Ness and Wintrob 1980; Phillips and Ano 2015). In addition, several authors proved that HIV + persons rarely return to religion as a form of coping with HIV-related trauma. In particular, Wanyama et al. (2007) showed that religious beliefs about HIV may cause fatalistic attitudes and resignation from treatment. Furthermore, Zou et al. (2009) proved that moral connotations usually associated with HIV infection can turn the religious community into a stigmatizing atmosphere for PLWH, which can lead them to withdraw from such a community. Mediation analysis revealed that relationship between religious fundamentalism and the PTSSs intensity was mediated by education level among participants. The higher the education, the lower the relationship between religious fundamentalism and the PTSSs intensity. Therefore, in the future investigation, it is necessary to verify the role of the education in religious fundamentalism—PTSSs relationship.

As noted above, religious coping may be related with positive mood and life satisfaction among PLWH (Lee et al. 2014) and may reduce depressive symptoms in PLWHA (Dalmida et al. 2013) and PTSSs in HIV-positive women (Brownley et al. 2015). On the other hand, Weber et al. (2012) showed that non-believers may experience psychological distress because of others’ negative perceptions. Returning to belief in the literal truth of the Bible and in the existence of a single, infallible set of religious truths may contribute to increasing subjective well-being and decreasing psychological distress related to HIV/AIDS infection. According to Carlucci et al. (2015), people who score high on religious fundamentalism scales tend to use religion to gain comfort, security and/or protection. Thus, anxiety, depression and PTSSs can motivate PLWHA to seek support in religion and strengthen their fundamentalist religious attitudes (Cotton et al. 2006). In terms of social stigma, turning to religion can be a powerful coping response when dealing with social rejection (Aydin et al. 2010). Compared with that of other European countries, religiousness in Poland is typically accompanied by a stable and relatively strong attachment to religious practices, such as church services, masses or encounters. According to the Public Opinion Research Centre (CBOS 2015), nearly 92% of all Poles declare that they are believers, and 87% of them are practicing religion. The hypothesis about increasing religious fundamentalism is being a way to reduce HIV-related trauma may be strongly supported in the Polish sample because of culturally inherited Catholicism, a strong attachment to own religion and the number of religious believers living in Poland. This hypothesis was not confirmed.

The results of the study show that satisfaction with life is negatively associated with PTSSs (see Table 2). The higher the PTSSs, the lower the life satisfaction in PLWHA. Satisfaction with life accounted for 30% of the variance of the trauma symptom intensity in PLWHA (see Table 3). Some major sources of this relationship may include PLWHA still experiencing social stigmatization that causes negative attitudes toward HIV-positive people and fear of contagion (Drewes and Kleiber 2014) and discrimination by families members (Adewuya et al. 2009; Gilbert and Walker 2010) and health professionals (Valenzuela et al. 2015). In other study, Cama et al. (2015) showed that PLWH appear to experience greater stigma related to taking HIV treatment than general stigma associated with HIV. HIV-related stigmatization seems to be a significant source of poor life satisfaction in PLWHA (Buseh et al. 2006; Heckman 2003). Other factors such as a life-threatening HIV infection and the unpredictable course of HIV/AIDS development may also lead to the occurrence of depression, anxiety and PTSSs among PLWHA (Lowther et al. 2014; Theuninck et al. 2010) and death anxiety following the PTSSs (Galinha and Pais-Ribeiro 2011; Routledge et al. 2010; Safren et al. 2003). On the other hand, Miller et al. (2012) showed that as the time since HIV diagnosis increased, PLWHA experienced less death anxiety.

Although satisfaction with life has a direct relationship with PTSSs, the connection between religious fundamentalism and satisfaction with life has not been confirmed. Religious fundamentalism may be found to be directly associated with trauma but not with satisfaction with life. Although Nell (2014) found no direct effect between religious fundamentalism and life satisfaction in his study, he did find a significant indirect effect in which the presence of life meaning mediated the relationship between religious fundamentalism and life satisfaction. This finding suggests that the relationship between religious fundamentalism and life satisfaction is indirect. A high level of fundamentalism, associated with clear and definite beliefs in an absolute truth, may lead believers to develop a meaning for life, which in turn enhances their life satisfaction. This result suggests the need to seek mediators between religious fundamentalism and satisfaction with life, especially in the elderly, who are more fundamentally religious, as indicated by the weak positive correlation between age and fundamentalism in the studied group (see Table 2).

The HIV/AIDS group experiences significantly lower life satisfaction compared with the HIV-positives but without AIDS (see Table 1). One of the reasons for this difference may be long-lasting infection in PLWHA group and the associated long-lasting psychological distress. However, the groups did not differ in PTSS level or the level of religious fundamentalism. The differences in the trauma symptom intensity between the two groups were also not demonstrated in an earlier study (Rzeszutek et al. 2012).

This study is not free of limitations. The study’s cross-sectional design does not allow for inferences about causation and the direction of causality. The sample was differentiated in terms of age, sex and phase of HIV infection development (HIV positive vs. HIV/AIDS). It does not include information about the possibility of previous traumatic events in participants’ lives that could have an impact on PTSS levels and assessment of satisfaction with life by PLWHA. The results suggest that future research should take into account not only religious fundamentalism but also the significance of social surroundings.

Despite these limitations, the results of this study provide a knowledge about the relationship between the PTSSs and satisfaction with life in PLWHA and may be a source of inspiration for further investigation and analysis referring to the relationship between religious fundamentalism and life satisfaction, taking mediating factors into an account.
